# Electrospinning of Magnetite–Polyacrylonitrile Composites for the Production of Oxygen Reduction Reaction Catalysts

**DOI:** 10.3390/polym15204064

**Published:** 2023-10-12

**Authors:** Al Mamun, Francisco José García-Mateos, Lilia Sabantina, Michaela Klöcker, Elise Diestelhorst, Ramiro Ruiz-Rosas, Juana María Rosas, José Rodríguez-Mirasol, Tomasz Blachowicz, Tomás Cordero

**Affiliations:** 1 Junior Research Group “Nanomaterials”, Faculty of Engineering and Mathematics, Bielefeld University of Applied Sciences and Arts, 33619 Bielefeld, Germany; 2 Department of Chemical Engineering, University of Malaga, Andalucia Tech., Campus de Teatinos, 29010 Málaga, Spain; 3 Faculty of Apparel Engineering and Textile Processing, Berlin University of Applied Sciences—HTW Berlin, 12459 Berlin, Germany; 4 Faculty of Engineering and Mathematics, Bielefeld University of Applied Sciences and Arts, 33619 Bielefeld, Germany; 5 Institute of Physics—CSE, Silesian University of Technology, 44-100 Gliwice, Poland; tomasz.blachowicz@polsl.pl

**Keywords:** electrospinning, ORR, fuel cells, PAN, magnetite, iron–nitrogen catalyst

## Abstract

In this study, electrospun carbon fiber electrodes were prepared by the carbonization of PAN–Fe_3_O_4_ electrospun fibers at 800 °C for their use as catalysts in the oxygen reduction reaction in an alkaline electrolyte. Magnetic nanofiber mats were fabricated using a needle-free electrospinning method by incorporating magnetic nanoparticles into a polymer solution. Electrochemical tests revealed that the oxygen reduction reaction (ORR) activity is optimized at an intermediate magnetite loading of 30% wt. These catalysts not only show better performance compared to their counterparts but also achieve high selectivity to water at low potentials. The onset and half-wave potentials of 0.92 and 0.76 V shown by these samples are only slightly behind those of the commercial Pt 20%-carbon black ORR catalyst. The obtained results point out that the electrospinning of PAN-Fe_3_O_4_ solutions allows the preparation of advanced N-Fe ORR catalysts in fibrillar morphology.

## 1. Introduction

The worldwide compromise to reach net zero emissions of greenhouse gases in 2050 demands the development of sustainable green energy storage solutions, such as metal–air batteries or fuel cells as complementary devices for the deployment of renewable energies and green hydrogen as an energy carrier [1,2]. For instance, alkaline fuel cells (FCs) are electrochemical devices that electrochemically oxidize hydrogen at the anode and reduce oxygen at the cathode, producing water [3]. The power and durability of FCs are related to the cathode performance, where the sluggish kinetics of the oxygen reduction reaction (ORR) originate most of the polarization losses. In order to overcome these limitations, the use of an alkaline electrolyte and platinum, which is highly active for the ORR and can reduce oxygen at high pH through the direct 4-electron process, is envisaged [4,5,6]. However, platinum presents several drawbacks, such as low stability and sensitivity to fuel crossover [7], that together with the scarcity and high costs hinder the large-scale commercial deployment of fuel cells, encouraging the research community to investigate alternative materials [8].

Given the low cost and high abundance of transition metals, they are gaining a great deal of attention as the ORR catalysts. Additionally, they show better stability in an alkaline medium compared to platinum-based electrocatalysts [9]. Single or mixed metal oxides and more specifically iron oxides are regarded as a platform to design novel ORR electrocatalysts due to their tunable composition that allows wise control of their physicochemical properties [10]. In this context, magnetite as an iron source can be viewed as an alternative to Pt-based electrocatalysts, since previous studies have reported that it has electrocatalytic activity towards the ORR [11]. However, such compounds show small surface area and intrinsic electrical conductivity, limiting their use as the ORR electrocatalysts. Consequently, metal oxides, even when prepared as nanosized materials, need to be supported over highly conductive materials to ease the transfer of electrons [12].

Electrospinning provides an outstanding tool for the preparation of polymer nanofibers. The advantage of using electrospinning does not lie only in obtaining nanosized fibers with controllable composition but also in the possibility of tuning the microstructure of the material by smart choice of the solution and spinning conditions. Roughly divided, there are two basic electrospinning processes, namely the needle-based electrospinning process and the needle-free electrospinning process [13]. The components of needle-free electrospinning are an upper wire, a lower wire, a carriage containing a polymer solution and a substrate for collecting the nanofibers, which are generated in a high-voltage field. The lower wire is coated with an electrospinning solution, Taylor cones are formed in the electrospinning process, and the resulting nanofibers are deposited onto a nonwoven substrate, such as polypropylene, located under the upper wire [14]. The needle-based electrospinning machine, on the other hand, consists of a pump, a syringe, a high-voltage power supply, a needle and a collector. The needle is connected to the positive pole of the power supply, while the collector is connected to the negative pole. The polymer solution is delivered from the syringe in a controlled manner, creating Taylor cones and seeding nanofibers onto the substrate [15]. It is also possible to synthesize metal-doped porous carbon fibers in a one-pot process by adding a metal salt to a spinnable polymer solution, which is later electrospun and carbonized [16]. Such a process has already produced a Pt-based electrocatalyst for methanol oxidation with excellent performance [17]. It should be noted that the resulting carbon nanofibers serve as a support for the dispersion of the active phase, thereby increasing the active surface area and, as a current collector, thereby mitigating the drawbacks of using metal oxides as the ORR electrocatalysts by themselves [18].

In addition, the carbonization of electrospun PAN nanofibers delivers the N-doping of the resulting carbon nanofibers [16], an additional positive feature since nitrogen groups promote wettability, conductivity and capacitance of carbon materials [17]. Interestingly, the ORR activity of nitrogen-containing carbon materials is enhanced when they are combined with iron, showing improved activity, a higher number of electrons transferred and better chemical stability [18]. Between them, the Fe-N4 has been proposed as the most promising active site for the ORR, constituting the most viable candidate to replace platinum in terms of activity and sustainability [19]. Hence, the electrospinning of PAN–magnetite mixtures could boost the activity of the resulting material not only by providing a simple procedure to disperse the actives sites but also by the positive interaction of PAN and magnetite to produce Fe-N pairs in a synergistic effect. 

In this work, the Fe-N ORR electrocatalysts are produced via the carbonization of Fe_3_O_4_-PAN electrospun nanofibers. Carbonization at 800 °C is performed to produce N-doped carbon nanofibers, which in turn leads to the formation of Fe-N active sites. The resulting Fe-N carbon nanofibers are tested as the ORR electrocatalysts in an alkaline electrolyte. Fe_3_O_4_-PAN nanofibers were previously characterized to determine their composition, surface properties and morphology. The effect of the Fe_3_O_4_ content in the resulting ORR catalysts was also addressed by modifying the amount of magnetite added to the PAN solution. To our knowledge, there is no report regarding the dispersion of magnetite in PAN nanofibers for the preparation of Fe-N-carbon nanofiber catalysts. Owing to the enhanced scalability of needless electrospinning, these results can pave the way for sustainable preparation of the Pt-free ORR catalysts.

## 2. Materials and Methods 

### 2.1. Fabrication Process of Electrospun Carbon Nanofiber Mats

**Figure 1:**
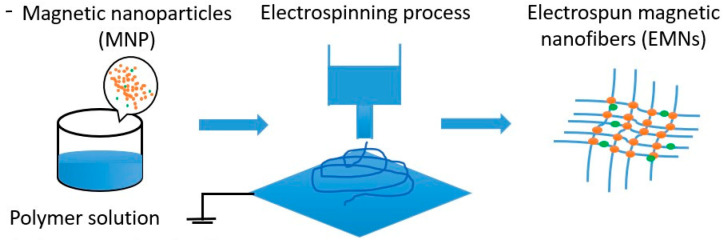
Schematic representation of the nanofiber manufacturing process in which magnetic particles are embedded. Adapted from [20] originally published under a CC-BY license.

To prepare the electrospinning solution, polyacrylonitrile (PAN) (X-PAN, Dralon, Dormagen, Germany) was dissolved in dimethyl sulfoxide (DMSO, min 99.9%, S3 Chemicals, Bad Oeynhausen, Germany) at room temperature using a magnetic stirrer for 1 h. Magnetic particles Fe_3_O_4_ (magnetite, particle size 50–100 nm, Merck KGaA, Darmstadt, Germany) were then manually stirred into the solution and subjected to ultrasonic treatment at 35 °C and a frequency of 37 kHz for 30 min. Figure 1 depicts a schematic representation of the fabrication of nanofibers with magnetic particles.

The first sample contained 25 wt.% Fe_3_O_4_ and 12 wt.% PAN, the second sample contained 30 wt.% Fe_3_O_4_ and 12 wt.% PAN, and the third sample contained 40 wt.% Fe_3_O_4_ and 10 wt.% PAN. Thus, the weight ratio of Fe_3_O_4_:PAN is 2.1:1, 2.5:1 and 4:1 for the corresponding samples. 

Nanofiber mats were produced using a needleless electrospinning machine Nanospider Lab (Elmarco, Liberec, Czech Republic), and nanofibers were collected on a nonwoven polypropylene substrate and afterwards separated. The following electrospinning parameters were employed: high voltage of 80 kV, nozzle diameter of 0.9 mm, carriage speed of 150 mm/s, mass-to-substrate distance of 240 mm and an electrode-to-substrate distance of 50 mm. The temperature and relative humidity in the electrospinning chamber were 22 °C and 32%, respectively. The electrospinning process continued for 20 min. 

Oxidative stabilization of the nanofiber mats was performed in a Nabertherm oven (Lilienthal, Germany). A typical stabilization temperature of 280 °C was used with a heating rate of 1 K/min and the final temperature was maintained for 1 h as was found to be the optimum temperature in our previous studies [21]. The Carbolite Gero furnace (Neuhausen, Germany) was used for the carbonization process of the nanofiber mats. A carbonization temperature of 800 °C was set at a heating rate of 10 K/min in a nitrogen gas flow of 100 mL/min (STP). The process was carried out in a nitrogen atmosphere followed by an isothermal treatment for 1 h after reaching the final temperature of 800 °C. 

Nanofiber diameters were determined using SEM micrographs and ImageJ software (version 1.53e, 2021, National Institutes of Health, Bethesda, MD, USA). To calculate the nanofiber diameter distribution, 100 fibers were measured. X-ray Photoemission Spectroscopy (XPS) analyses were performed in a PHI 5000 VersaProbe II equipment, using a monochromatic Al X-ray source.

The FlexAFM Axiom (Nanosurf, Liestal, Switzerland) and a confocal laser scanning microscope (CLSM), VK-8710 (Keyence) were utilized for optical analyses. Surface morphology was further examined using an SDD-X-Max Extreme (80 mm^2^ SDD detector, Oxford Instruments, Wiesbaden, Germany) scanning electron microscope (SEM) and energy dispersive X-ray spectroscopy (EDS). Fourier transform infrared (FTIR) spectroscopy was conducted using an Excalibur 3100 (Varian, Inc., Palo Alto, CA, USA) with a spectral range of 4000 cm^−1^ to 700 cm^−1^. Additionally, 32 scans were averaged, and atmospheric noise was corrected.

### 2.2. Electrochemical Testing Procedure

An SP-200 potentiostat (Biologic, Seyssinet-Pariset, France) was employed to conduct the electrochemical analysis of the electrocatalysts in a 3-electrode cell arrangement. In this setup, a platinum wire and a saturated calomel electrode (SCE) were utilized as the counter and reference electrodes, respectively. The working electrode was comprised of an OrigaTrod (Origalis) rotating disk electrode (RDE) equipped with a tip of glassy carbon (GC) having a diameter of 5 mm.

To prepare the working electrode, a solution with a concentration of 1 mg/mL ink was formulated. This solution was created by combining 5 mg of the sample with 5 mL of a solution composed of deionized water, isopropanol and Nafion© (Sigma-Aldrich, St. Louis, MO, USA), using weight ratios of 5:1:0.5. The resultant mixture underwent sonication in an ultrasound bath for a duration of 20 min. Subsequently, the GC electrode was loaded with the sample through the application of 20 μL of the ink onto the surface of the GC electrode. The GC electrode was then dried at a temperature of 110 °C, resulting in an electrode loading of approximately 100 μg/cm^2^.

Tests to evaluate the electrochemical activity of the sample in oxygen reduction reaction (ORR) were carried out at a temperature of 25 °C within an alkaline electrolyte solution containing 0.1 M KOH. The behavior of the working electrode was analyzed after a stabilization treatment of 20 cycles of cyclic voltammetry (CV) at a scan rate of 50 mV/s. These cycles were conducted within the voltage range of 0 to 1.2 V versus the reversible hydrogen electrode (RHE) using an electrolyte solution saturated with nitrogen (N_2_). From that point, linear sweep voltammetry (LSV) experiments were performed in two separate electrolyte environments, one saturated with nitrogen (N_2_) and the other saturated with oxygen (O_2_). These tests encompassed the voltage range from 0 to 1 V and were executed at a scan rate of 5 mV/s, while varying the rotational speed of the electrode (ranging from 400 to 2025 rpm). The LSV recorded in saturated nitrogen atmosphere was used as baseline to obtain the oxygen reduction reaction (ORR) LSV profile. The number of electrons transferred during ORR, represented as ‘*n*’, was determined through the utilization of the Kouteky–Levich (KL) equation. The equation employed for this determination was as follows:

(1) $$\frac{1}{j}=\frac{1}{j_{K}}+\frac{1}{J_{L}}=\frac{1}{j_{K}}+\frac{w^{-1/2}}{0.62\cdot D_{O2}^{2/3}\cdot C_{O2}\cdot v^{-1/6}\cdot F\cdot n}$$

where *j_K_* and *j_L_* are the kinetic and mass transfer limited current densities, *j* is the experimentally observed current density, *w* is the angular velocity, *D_O_*_2_ is the diffusion coefficient of oxygen, *C_O_*_2_ is the bulk concentration of O_2_, v is the kinematic viscosity of the electrolyte, and *F* is the Faraday constant. 

The electrochemical response and ORR activity of commercial 20% platinum on Vulcan XC72 catalyst (PtC, Sigma-Aldrich) were also analyzed using the same protocol for comparison purposes.

## 3. Results

### 3.1. Characterization of Samples

In this study, the weight percentages of Fe_3_O_4_ and PAN in nanofiber mats were varied to investigate their impact on the stabilization and carbonization yields. Table 1 shows the stabilization and carbonization yields of the samples.

The samples included 25 wt.% Fe_3_O_4_, 30 wt.% Fe_3_O_4_ and 40 wt.% Fe_3_O_4_, expressed as weight ratios. The stabilization and carbonization yields were expressed as weight percentages, and the total yield was also determined for each sample. The results showed that the nanofiber mat with 25 wt.% Fe_3_O_4_ exhibited the highest overall yield of 55.4%, with a stabilization yield of 89.5% and a carbonization yield of 61.9%. The sample containing 30 wt.% Fe_3_O_4_ demonstrated a stabilization yield of 79.2%, while the carbonization yield was not measurable due to an insufficient material yield after carbonization. 

The nanofiber mat with 40% Fe_3_O_4_ exhibited the lowest overall yield of 20.2%, with a stabilization yield of 93.7% and a carbonization yield of 21.5%. These results suggest that the weight percentages of Fe_3_O_4_ and PAN may have a significant effect on the stabilization and carbonization yields of nanofiber mats. In particular, the concentration of PAN in the spinning solution has a discernible effect on the resulting carbonization yield, rendering PAN an important precursor material in the synthesis of carbon nanofibers.

**Figure 2:**
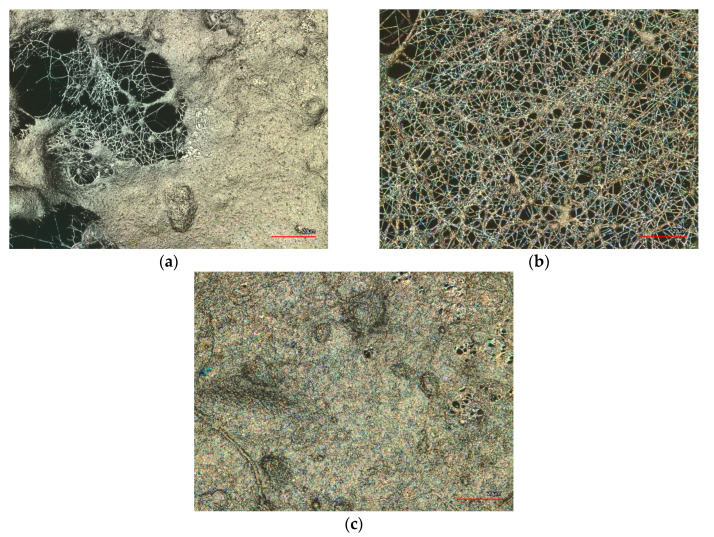
Confocal laser scanning microscope (CLSM) images of the magnetic nanofiber mats: (**a**) 25 wt.% magnetite/PAN nanofiber mat; (**b**) 30 wt.% magnetite/PAN nanofiber mat; (**c**) 40 wt.% magnetite/PAN nanofiber mat. The scale bars indicate 20 μm.

The morphology of nanofiber mats containing different weight percentages of Fe_3_O_4_ nanoparticles was investigated by using confocal laser scanning microscopy (CLSM). Figure 2 shows the surface morphology of the nanofiber mats with 25 wt.%, 30 wt.% and 40 wt.% Fe_3_O_4_. 

**Figure 3:**
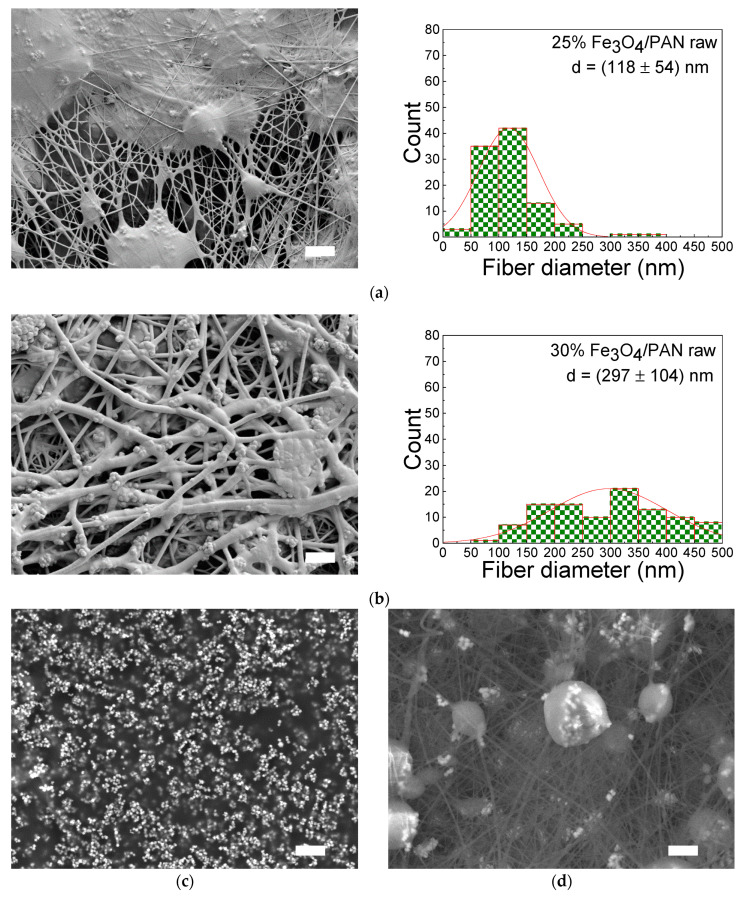
Scanning electron microscope (SEM) micrographs and distribution of the fiber diameters of (**a**) 25 wt.% magnetite/PAN nanofiber mat; (**b**) 30 wt.% magnetite/PAN nanofiber mat; (**c**) 40 wt.% magnetite/PAN nanofiber mat (without the distribution of the fiber diameters); (**d**) a nanofiber mat containing 25 wt.% of magnetite and displaying beads. The scale bars indicate 2 μm.

The CLSM images show that the nanofiber mats with 30 wt.% Fe_3_O_4_ have nanofibers with some beads, while the mats with 25 wt.% and 40 wt.% Fe_3_O_4_ have larger membrane areas. The variations in the surface morphology of the nanofiber mats are likely attributed to the differences in the magnetite and PAN concentrations. Furthermore, the differences in the surface morphology are not always comprehensible, as discussed in the study by Wortmann et al. [22]. This implies that the nanofiber mats containing 30 wt.% Fe_3_O_4_ exhibit a more consistent morphology compared to that of the nanofiber mats with 25 wt.% and 40 wt.% Fe_3_O_4_, primarily due to their higher nanofiber density (see Figure 3). The beads observed in the nanofiber mats with 30 wt.% Fe_3_O_4_ (see Figure 3b) could be due to less agglomeration of Fe_3_O_4_ nanoparticles, which can affect the overall performance of the nanofiber mats as catalysts for the ORR. The mats with 25 wt.% (see Figure 3a) and 40 wt.% Fe_3_O_4_ (see Figure 3c), on the other hand, have a more irregular morphology with larger membrane areas, which could provide a larger surface area for catalytic reactions to take place. In Figure 3a, a nanofiber mat containing 25 wt.% of magnetite is depicted using a scattering electron SEM technique. To enhance the visibility of the beads, an additional SEM image (Figure 3d) was incorporated, captured using a variable pressure electron SEM technique. It is presumed that the nanoparticles accumulate in beads, and numerous beads are also evident in the nanofiber mat containing 30 wt.% of magnetite. Overall, the SEM images provide detailed views of the nanofiber mats and the effects of magnetic particle concentration on fiber diameter and morphology. The SEM images confirm that the nanofiber mats with 30 wt.% magnetic particles have more visible fibers than membrane areas on the surface compared to the mats with 25 wt.% and 40 wt.% magnetic particles. However, the reasons for membrane areas are diverse. For instance, this may be attributed to the incomplete evaporation of the solvent during the electrospinning process, resulting in the formation of membrane areas. Making general statements based on a limited number of samples is problematic, as critically pointed out by Wortmann et al. [22].

The average fiber diameter of the nanofibers is (118 ± 54) nm with 25 wt.% magnetic particles, indicating relatively thin fibers. However, the average fiber diameter of the nanofibers increases significantly to (297 ± 104) nm with 30 wt.% magnetic particles, indicating a noticeable increase in fiber thickness compared to the 25 wt.% sample. This increase in fiber diameter is likely due to the presence of a higher concentration of magnetic particles, which could affect the electrospinning process and alter the resulting fiber morphology. In electrospinning, the diameter of the nanofibers is affected by various factors such as the concentration of the polymer solution, the viscosity of the solution, the electrical conductivity of the solution, the applied voltage and the distance between the needle and the collector [23]. It is possible that the presence of magnetic particles could affect the solution viscosity, resulting in thicker fibers. Additionally, the magnetic particles could also affect the surface tension and charge density of the solution, altering the electrospinning process and fiber morphology. Overall, the increase in fiber diameter with increasing magnetic particle concentration suggests that optimizing the electrospinning process parameters is crucial for producing nanofiber mats with desired properties [24].

**Figure 4:**
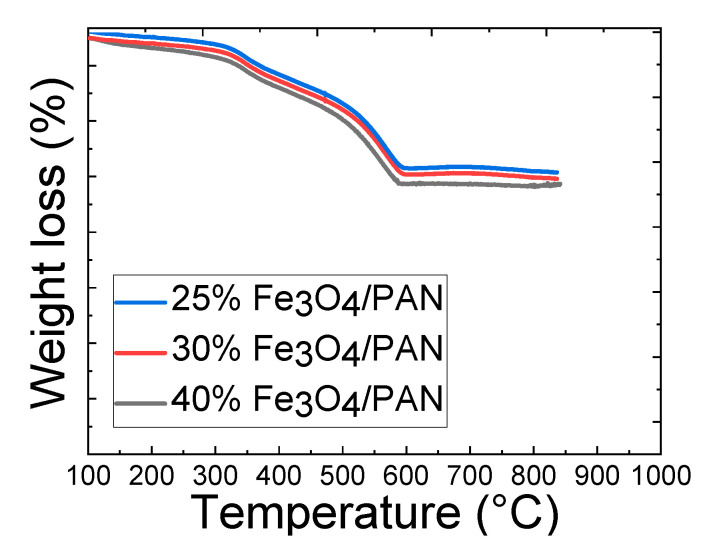
Thermogravimetric analysis (TGA) in air of 25 wt.%, 30 wt.% and 40 wt.% magnetite/PAN nanofiber mats.

Thermogravimetric analysis (TGA) is a technique that measures the weight change of a material as it is heated or cooled under controlled conditions. The TGA diagram in Figure 4 shows the weight loss of the magnetic nanofiber mats as a function of temperature.

The gradual weight loss observed in the TGA diagram for all the samples is likely due to the decomposition and/or oxidation of the polymer matrix and the thermal degradation of the magnetite nanoparticles embedded within the nanofiber mats. The thermal degradation of the fibers seems to be relevant from 300 °C, therefore the temperature for the stabilization treatment was set at 280 °C in order to avoid the decomposition of the polymeric matrix. 

**Figure 5:**
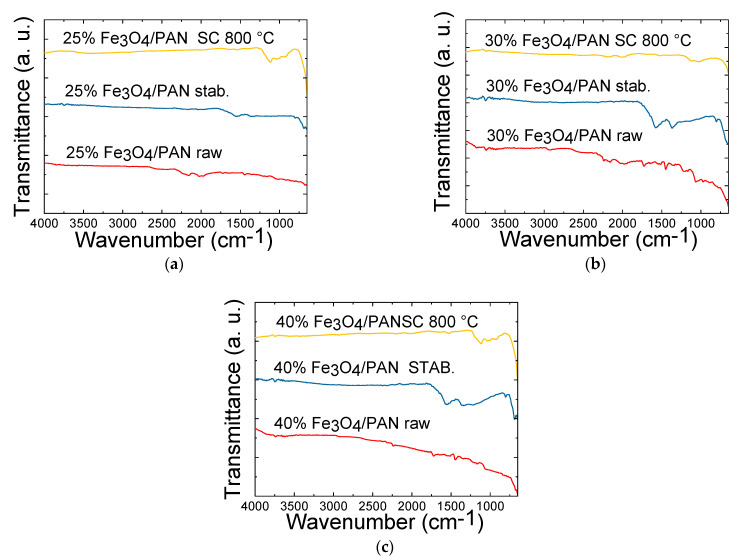
Fourier-transform infrared spectroscopy (FTIR) measurements of PAN/magnetite samples after electrospinning, stabilization at 280 °C and carbonization at 800 °C: (**a**) 25 wt.% magnetite/PAN nanofiber mat; (**b**) 30 wt.% magnetite/PAN nanofiber mat; (**c**) 40 wt.% magnetite/PAN nanofiber mat. The lines are vertically offset for clarity.

Figure 5 displays the FTIR spectra of PAN/magnetite nanofiber mats, which provide chemical information about the samples. 

The spectra exhibit characteristic peaks corresponding to PAN, including CH_2_ bending and stretching vibrations at 2938 cm^−1^, 1452 cm^−1^ and 1380 cm^−1^ (see Figure 5a–c). Additionally, stretching vibrations of the nitrile group at 2240 cm^−1^ and the carbonyl stretching peak at 1731 cm^−1^ are visible in all spectra of the PAN/magnetite nanofiber mats with 25 wt.%, 30 wt.% and 40 wt.% magnetic particles after electrospinning. Comparing the spectra of the nanofiber mats after electrospinning to those obtained after stabilization process, a peak at ca. 1600 cm^−1^ appeared, while the strengths of the peaks at 2938, 2240 and 1452 cm^−1^ diminished. These changes are attributed to the cyclization and dehydrogenation processes undergone by the fibers, confirming that they are indeed stabilized [25]. Similar behavior is observed for all the samples, no matter the amount of magnetic nanoparticles that are added in the formulation. After carbonization, the mats lost most of the infrared-active functional groups owing to the devolatilization, aromatic condensation and carbonization reactions. Consequently, the FTIR profiles of the carbonized samples at 800 °C show a lower number of and less intense peaks, apart from those related to C=N and C=C−H bonds [26].

**Figure 6:**
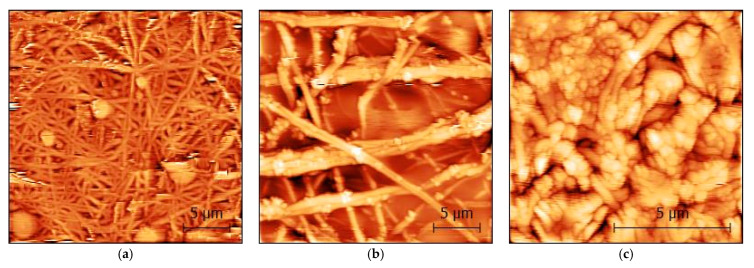
Atomic force microscope (AFM) micrographs of carbonized (**a**) 25 wt.% magnetite/PAN nanofiber mat; (**b**) 30 wt.% magnetite/PAN nanofiber mat; (**c**) 40 wt.% magnetite/PAN nanofiber mat. The scale bars indicate 5 μm.

Figure 6 shows the atomic force microscopy images of carbon nanofiber mats with varying percentages of magnetite nanoparticles. The use of AFM allows for a more detailed examination of the surface features and can provide insight into the factors that contribute to the observed deviations from the desired morphology. This information can then be used to optimize the synthesis and processing conditions to achieve the desired surface properties and improve the catalytic performance of the nanofiber mats for the ORR.

While the carbonized 25 wt.% and 30 wt.% magnetite/PAN nanofiber mats still preserve nanofibrillar morphology, the samples obtained with 40 wt.% magnetic particles show clear deviations from the desired nanofiber morphology. This could be due to the presence of agglomerated nanoparticles or other factors that affect the electrohydrodynamic forces that governs the fate of the jet of liquid ejected from the Taylor cone, such as excessive electrical conductivity or viscosity of the solution, favoring the preponderance of electrospray over electrospinning. 

**Figure 7:**
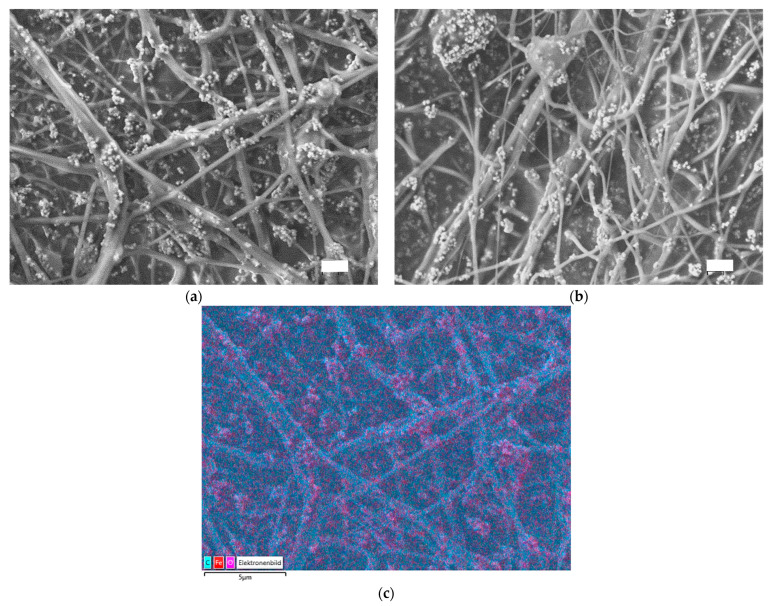
Scanning electron microscope (SEM) micrographs of 30 wt.% magnetite/PAN nanofiber mat: (**a**) after electrospinning; (**b**) energy dispersive X-ray spectroscopy (EDS) showing magnetite in red color; (**c**) stabilized at 280 °C and carbonized at 800 °C. The scale bars indicate 2 μm (**a**,**b**).

The morphologic changes introduced by the stabilization and carbonization of 30 wt.% magnetite/PAN nanofiber were tracked by scanning electron microscope (SEM). The micrograph of the prepared nanofiber mat (Figure 7a) and the corresponding SEM image of the sample (Figure 7b) are compared to those of the sample stabilized at 280 °C and carbonized at 800 °C (Figure 7c). No relevant changes are introduced in the morphology of the mats after each treatment step. Moreover, the EDS map confirms that the bright spheres are mainly composed of iron. The distribution of nanoparticles is important because it affects the efficiency and effectiveness of the catalyst. If the nanoparticles are not well distributed, the catalytic performance may be compromised. The obtained images point out that the adequate distribution of the magnetic nanoparticles in the starting sample is well preserved after carbonization (Figure 7c).

**Figure 8:**
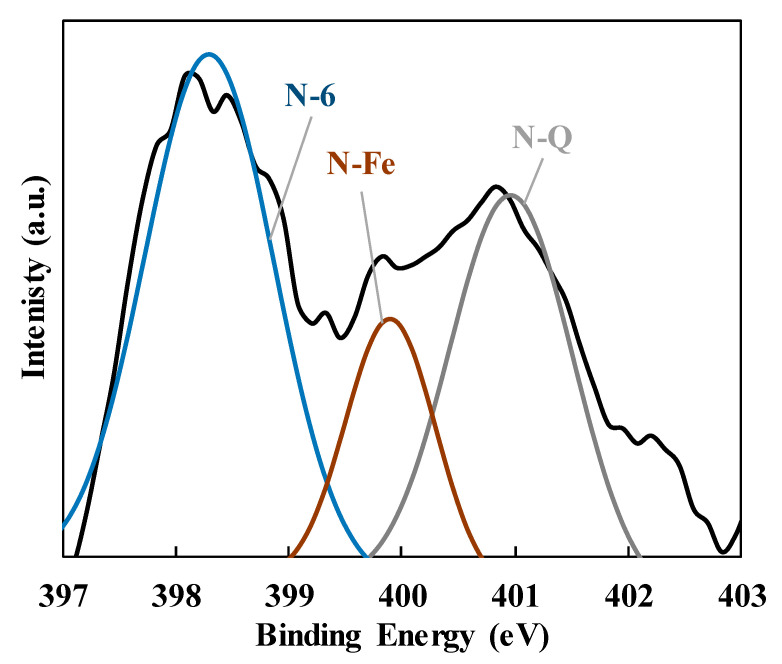
N1s XPS photoemission region of the Fe30 sample. Black line: experimental profile. N-6: pyridinic nitrogen. N-Q: quaternary nitrogen. N-Fe: nitrogen coordinated to iron.

The XPS analyses confirmed the presence of nitrogen and iron on the surface of the samples, showing concentrations of 3.2 and 8.9 wt.%, respectively. The N1s XPS photoemission regions recorded for the Fe30 sample are included in Figure 8. The N1s region presents two broad peaks that were assigned to the presence of pyridinic nitrogen (398.3 eV), N-Fe sites in pyrrolic configuration (399.7 eV) and quaternary nitrogen (400.9 eV) [27,28]. This result confirms that N-Fe active sites can be obtained through the carbonization of magnetite–PAN electrospun fibers.

### 3.2. Oxygen Reduction Reaction Tests

**Figure 9:**
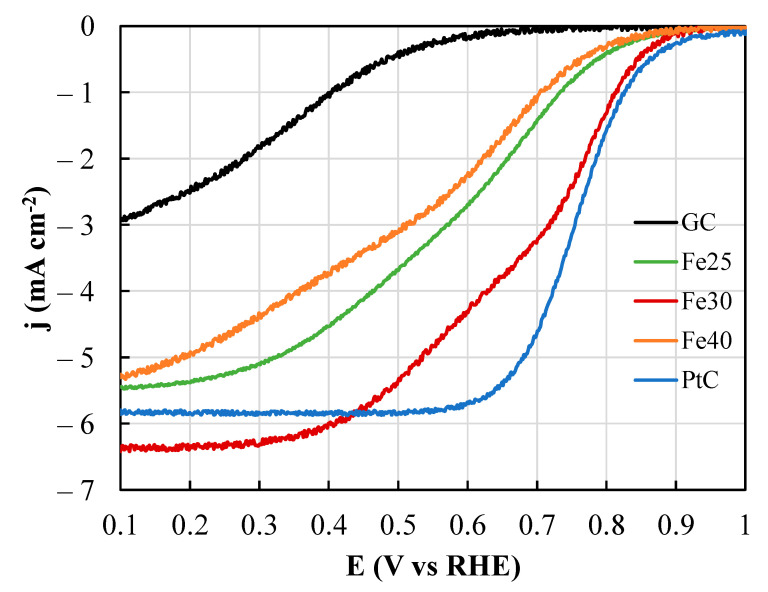
Corrected linear sweep voltammetry of glassy carbon (GC), Fe25, Fe30, Fe40 and Vulcan XC72-20%Pt (PtC) samples at 5 mV s^−1^ in O_2_-saturated 0.1 M KOH. Rotating speed: 1600 rpm.

Figure 9 illustrates the linear sweep voltammetry (LSV) plots obtained at 1600 rpm speeds for the three samples. The response of the bare glassy carbon surface and that of the commercial 20 wt.% Pt/C Vulcan catalyst are also included for comparison purposes. All the prepared samples showed higher catalytic activity compared to the glassy carbon surface, confirming the presence of electroactive Fe-N groups. The LSV profiles of the samples demonstrate a two-step waveform, with the second wave initiating around 0.7 V for the Fe30 catalyst. This behavior has been attributed to a mixed 2 + 2 electron ORR mechanism [29]. In this mechanism, oxygen undergoes reduction at elevated potentials to yield hydrogen peroxide, involving 2 electrons. Subsequently, hydrogen peroxide is further reduced to water at moderate and low potentials (as observed in the second wave of the LSV plot), ultimately generating the final 2 electrons. In addition, both the Fe25 and Fe40 samples exhibit a tilted LSV profile, not being able to reach diffusion-limited currents, which are indeed presented by Fe30 starting from 0.35 V. The profiles within the transitional region of the former samples exhibit smaller slopes compared to that of Fe30, indicating the lower electrode resistance of the latter catalyst. Finally, while Fe25 and Fe40 share similar onset potentials, Fe30 displays a higher onset potential, indicative of the enhanced activity of this electrocatalyst, almost matching that of the commercial catalyst. This particular behavior might be explained by the changes in electroactive areas. As previously discussed, the excess of iron oxide compromises the continuity and occurrence of fibers in the membrane, which is translated into a much lower surface-to-volume ratio. In addition, the carbon electrodes turned brittle for Fe40, being difficult to handle and deploy on the RDE.

**Figure 10:**
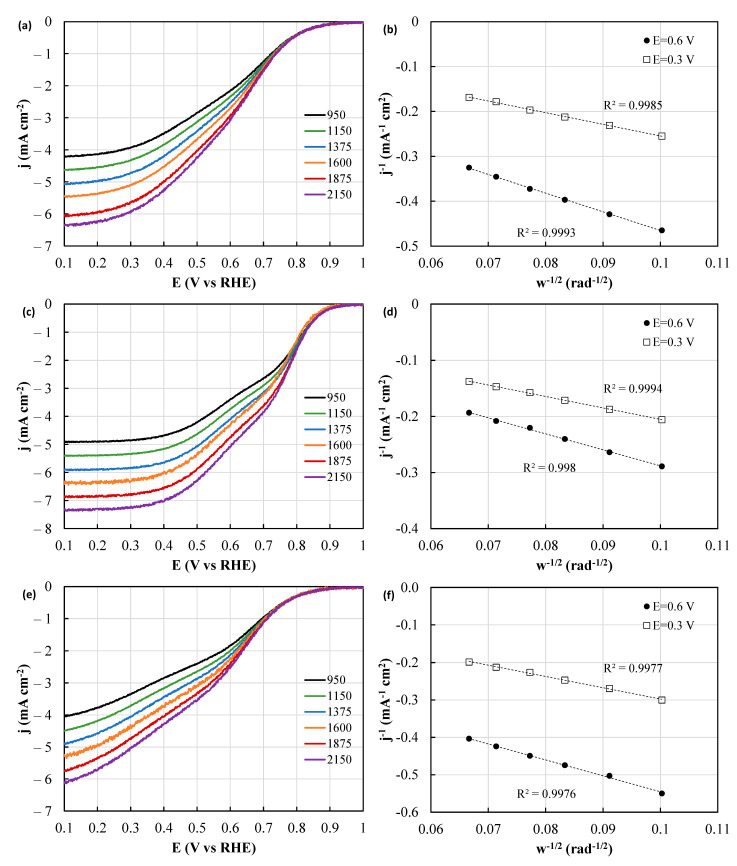
Linear sweep voltammetry of (**a**) Fe25, (**c**) Fe30 and (**e**) Fe40 recorded at 5 mV s^−1^ and different rotating speeds in O_2_-saturated 0.1 M KOH. Kouteky–Levich plots of (**b**) Fe25, (**d**) Fe30 and (**f**) Fe40 samples at 0.3 and 0.6 V vs. RHE.

The catalytic performance associated with the oxygen reduction reaction (ORR) involves not only the onset potential and half-wave potentials, but more importantly, the number of electrons transferred, which should be close to 4 in order to achieve near full selectivity towards water and maximize the energy generation. The determination of this parameter was performed by applying the KL method at low (0.3 V) and intermediate (0.6 V) potentials to the LSV curves recorded at different rotating speeds between 400 and 2150 rpm (Figure 10). As anticipated, as the rotation rate increases, all catalysts exhibit an augmented ORR limiting current. Nonetheless, the increase in the specific current varies depending on the sample and on the potential, pointing out that different number of electrons are being transferred. It can be seen that the KL plots show linear behavior in all the cases, confirming the validity of the proposed method for the determination of the n (n, calculated using the slope of the KL plot as elaborated in the experimental section). In order to ease the comparison between the different samples, the onset and half-wave potentials, along with the number of electrons transferred at 0.3 and 0.6 V (determined from the slope of the KL plots) were calculated from the LSV profiles and gathered in Table 2. Notably, the determination of the electron count is most accurately achieved using a rotating ring disk electrode [30], and, to provide proper context, the KL analysis results of PtC are also incorporated.

It becomes evident that the onset potential, a crucial indicator of the ORR activity, follows the sequence Fe30 > Fe25 > Fe40. The superior performance of Fe30 persists at the half-wave potential. Additionally, the Kouteky–Levich (KL) plots unveil that in Fe25 and Fe40, the oxygen reduction predominantly follows the two-electron transfer pathway at intermediate voltages, while in Fe30, the ORR seems to proceed through the mixed 2 + 2 electron mechanism at intermediate voltage, reaching the 4-electron mechanism at low potentials. Surprisingly, the number of transferred electrons surpasses that of PtC at 0.3V (Table 2). This feature has been already found for other catalytic systems with a large surface area and is attributed to the higher roughness of the electrode (i.e., the effective area is larger than the geometric area), which certainly produce deviations from the KL assumptions when determining the number of transferred electrons [31,32].

The remarkable ORR performance of Fe30 exhibits a catalytic activity comparable to that achieved with Fe-containing amino acid-doped pyrolyzed rGO, as reported in our prior study [28]. To further contextualize the activity of this catalyst, comparisons were made against Fe-N-C catalysts synthesized using different supports. In this context, the ORR performance of Fe30 is at least comparable in terms of the onset and half-wave to that of the next catalysts: the Fe-N-C catalyst produced by pyrolysis of Fe_3_O_4_ nanoparticles covered with polyaniline and pyrolyzed at 1000 °C [33]; the one obtained from the pyrolysis of hydrothermal carbons doped with histidine and iron nitride and subsequently etched with ammonia [34]; the Fe-N doped template carbons synthesized through a hard template method involving histidine and iron (III) mixtures [35]. The ORR activity is slightly lower than the one shown by N-doped hierarchical porous carbon obtained by the carbonization of sucrose on top of SiO_2_ spheres in the presence of NH_3_ and decorated with Fe_3_O_4_ particles [36]; however, the proposed methodology involves fewer steps and avoids the use of toxic and dangerous reagents, such as NH_3_ or HF.

## 4. Discussion

The use of catalysts for the oxygen reduction reaction (ORR) is critical in various fields such as energy conversion and storage, including fuel cells, metal–air batteries and supercapacitors. The ORR is a chemical reaction that occurs when oxygen reacts with a reducing agent, such as a fuel, to produce water and energy. However, the reaction can be slow and inefficient without the use of a catalyst, which can increase the rate of reaction and reduce the amount of energy required to drive the reaction. There has been a significant amount of research in recent years focused on developing effective catalysts for the ORR. Some of the most commonly used catalysts include platinum (Pt) and its alloys due to their high activity and stability. However, the high cost and limited availability of Pt make it less desirable for large-scale applications. As a result, there is a need for alternative catalysts that are cost-effective, stable and exhibit high activity. Carbon-based materials, including carbon nanofiber mats, have shown great potential as alternative catalysts for the ORR due to their large surface area, electrical conductivity and chemical stability. In recent years, research has been focused on developing carbon magnetic nanofiber mats as catalysts for the ORR. These materials combine the benefits of carbon nanofiber mats with the magnetic properties of iron oxide, which can enhance their activity as catalysts. Several studies have investigated the properties and performance of carbon magnetic nanofiber mats as catalysts for the ORR. These studies have explored various factors that can affect the performance of the mats, such as the ratio of magnetic material to polymer, the production process and the morphology of the mats. Some studies have also investigated the use of other magnetic materials, such as cobalt and nickel, in carbon nanofiber mats as alternative catalysts for the ORR.

Overall, the development of effective and efficient catalysts for the ORR is critical for advancing various technologies in the field of energy conversion and storage. Carbon magnetic nanofiber mats represent a promising alternative to traditional catalysts, and continued research in this area has the potential to lead to significant advancements in the field. The morphological and chemical properties of carbon magnetic nanofiber mats can have a significant impact on their performance as catalysts for the ORR. Morphologically, the surface area, pore size distribution and surface roughness of the nanofiber mats can affect the activity and stability of the catalyst. A large surface area can provide more active sites for the reaction to occur, while narrow pore size distribution can improve mass transport of reactants and products to and from the active sites. Surface roughness can also play a role in promoting the formation of intermediate species and increasing the contact area between the catalyst and reactants. One of the main findings of this study is that intermediate Fe loadings are needed to optimize the catalytic activity. This result can be connected to the Sabatier principle, which asserts that the optimal catalyst should exhibit a binding affinity for reaction intermediates that is neither excessively strong nor excessively weak [37]. Excessive or low iron loading might lead to unbalanced binding between the active sites and oxygen.

Chemically, the composition and surface functionalization of the nanofiber mats can affect their activity and selectivity as catalysts. For example, doping the carbon matrix with heteroatoms, such as nitrogen or sulfur, can modify the electronic properties of the carbon surface and improve the activity and selectivity of the catalyst. Additionally, surface functionalization with various functional groups can alter the surface charge and polarity of the catalyst, affecting its interaction with reactants and intermediates. The presence of magnetic nanoparticles within the carbon nanofiber mats can also influence the catalyst performance. The magnetic nanoparticles can improve the dispersion and stability of the catalyst and enhance the electron transfer and mass transport of reactants and intermediates. Therefore, it is crucial to consider the morphological and chemical properties of carbon magnetic nanofiber mats when designing and optimizing them as catalysts for the ORR. By controlling these properties, it is possible to improve the activity, stability and selectivity of the catalyst, making it more efficient and effective for various applications.

## 5. Conclusions

The results reported in this work confirm that it is possible to reach similar ORR activity than that of a commercial catalyst, using carbonized PAN-Fe_3_O_4_ composites. This result alone is not surprising, since the outstanding activity of the Fe-N catalysts have been proven in the past [35,36]. However, the successful deployment of such active centers in electrospun membranes while keeping high and selective ORR catalytic activity is a novel finding that increases their potential applications in electrochemical devices. Note that the self-standing configuration of the mats prepared herein presents notable advantages from the point of view of the electrode processing. Using these mats as electrodes would make it possible to avoid additional preparation steps for powdered catalysts, such as mixing with binders and conductivity promoters, preparing an ink, depositing the catalytic phase on top of the gas diffusion layer, developing a controlled dry process, etc. The preparation of these carbonized membranes by electrospinning could be also complemented by adding a final electrospray/electrospinning layer of an ionomer that would conform the membrane, paving the way for the incorporation of electro-processing in the production of membrane-electrode assemblies with improved performance in fewer preparation steps.

## Figures and Tables

**Table 1:** Overview of stabilization and carbonization yields of the samples.

Samples	Stabilized (wt.%)	Carbonized (wt.%)	Overall Yield (wt.%)
Fe25 (25 wt.% Fe_3_O_4_)	89.5	61.9	55.4
Fe30 (30 wt.% Fe_3_O_4_)	79.2	-	-
Fe40 (40 wt.% Fe_3_O_4_)	93.7	21.5	20.2

**Table 2:** Onset potential and number of transferred electrons of Fe25, Fe30 and Fe40 samples.

Samples	Onset Potential at 0.1 mA cm^−2^	Half-Wave Potential at j_L_ = 0.5 mA cm^−2^	Number of Electrons (at 0.6 V vs. RHE)	Number of Electrons (at 0.3 V vs. RHE)
Fe25	0.888 V	0.596 V	2.18	3.44
Fe30	0.920 V	0.716 V	3.08	4.37
Fe40	0.872 V	0.554 V	2.04	2.95
PtC	0.988 V	0.753 V	4.03	4.01

## Data Availability

All data obtained in this study are part of this paper.
